# EP01 Post COVID-19 reactive arthritis

**DOI:** 10.1093/rap/rkaa052

**Published:** 2020-11-03

**Authors:** Rosemary Waller, Elizabeth Price, Sara Carty, Azeem Ahmed, David Collins

**Affiliations:** r1 Great Western NHSFT, Swindon, United Kingdom; r2 Great Western NHFT, Swindon, United Kingdom

## Abstract

**Case report - Introduction:**

We present what we believe to be the first reported case of post COVID-19 reactive arthritis, in a previously medically well 16-year-old with no past or family history of inflammatory arthritis.

**Case report - Case description:**

Our patient was a previously medically fit 16-year-old of Caucasian origin who tested positive for COVID-19 in late March 2020. She developed with a 4-day illness with fever, cough, and myalgia from which she made a full and uncomplicated recovery.

Ten days later she developed a new erythematous itchy rash on her legs, trunk, and face and a progressive polyarthralgia affecting her MCPs, wrists, shoulders, hips, and knees. The rash typically lasted for 2 days at one site and was non-scarring. This was associated with a low-grade fever. There were no associated mouth ulcers, photosensitivity, alopecia, Raynaud’s, GI disturbance or respiratory symptoms. She had no relevant family history of autoimmunity, psoriasis or inflammatory bowel disease or travel history and had been prescribed no new medications.

On examination, she had an erythematous rash on the face in a non malar distribution. She had multiple tender joints without definite synovitis. Cardiovascular, respiratory, gastroenterology and neurological examinations were unremarkable.

Investigations revealed a normal full blood count and CRP<1 with normal liver and renal function tests. Her urinalysis was unremarkable. Immunology was negative for ANA, ANCA and rheumatoid factor. Immunoglobulins were normal. Two weeks later her symptoms were fully resolved.

**Case report - Discussion:**

Coronaviruses are single-stranded RNA viruses with nearly 30 strains recognised to infect humans. They induce both an innate and adaptive immune system response. It is hypothesised that a dysregulated innate system response, leading to a prolonged adaptive response triggers damaging inflammation and a potential cytokine storm. This is associated with poor outcome during primary viral infection. Variations in this immune response, with different underlying HLA genotypes could lead to other post infectious immune mediated phenomena, such as Paediatric Multisystem Inflammatory Syndrome - Temporally associated with COVID-19.

There is a European registry collating data about patients with known rheumatic diseases who are admitted with COVID-19. There is emerging data regarding Paediatric Inflammatory Multisystem Syndrome - Temporally associated with SARS-CoV-2 (PIMS-TS). There is a growing suggestion that a subgroup of patients is developing a COVID-19 associated post viral fatigue syndrome. We suggest that a registry to collect information on de novo autoimmune diseases presenting post COVID-19 is also commenced.

**Case report - Key learning points:**

COVID-19 infection is associated with a wide variety of sequalae, including rheumatological ones.

Classic post viral Reactive arthritis has been seen. A registry to collect information on de novo autoimmune presentations would be highly informative.

